# Spontaneous imbibition in fractal tortuous micro-nano pores considering dynamic contact angle and slip effect: phase portrait analysis and analytical solutions

**DOI:** 10.1038/s41598-018-21002-y

**Published:** 2018-03-02

**Authors:** Caoxiong Li, Yinghao Shen, Hongkui Ge, Yanjun Zhang, Tao Liu

**Affiliations:** 1 Unconventional Natural Gas Institute, China University of Petroleum, Beijing, 102249 China; 2 School of Engineering Science, University of Chinese Academy of Sciences, Beijing, 100049 China; 3 China University of Petroleum, Karamay campus, Karamay, Xinjiang 834000 China; 4 Oil & Gas Technology Research Institute, Changqing Oilfield Company, Xi’An, 710018 China

## Abstract

Shales have abundant micro-nano pores. Meanwhile, a considerable amount of fracturing liquid is imbibed spontaneously in the hydraulic fracturing process. The spontaneous imbibition in tortuous micro-nano pores is special to shale, and dynamic contact angle and slippage are two important characteristics. In this work, we mainly investigate spontaneous imbibition considering dynamic contact angle and slip effect in fractal tortuous capillaries. We introduce phase portrait analysis to analyse the dynamic state and stability of imbibition. Moreover, analytical solutions to the imbibition equation are derived under special situations, and the solutions are verified by published data. Finally, we discuss the influences of slip length, dynamic contact angle and gravity on spontaneous imbibition. The analysis shows that phase portrait is an ideal tool for analysing spontaneous imbibition because it can evaluate the process without solving the complex governing ordinary differential equations. Moreover, dynamic contact angle and slip effect play an important role in fluid imbibition in fractal tortuous capillaries. Neglecting slip effect in micro-nano pores apparently underestimates imbibition capability, and ignoring variations in contact angle causes inaccuracy in predicting imbibition speed at the initial stage of the process. Finally, gravity is one of the factors that control the stabilisation of the imbibition process.

## Introduction

Shale has relatively low permeability and porosity with most hydrocarbon stored in its tight matrix. Hydraulic fracturing is commonly used during the shale gas development process. Tons of fracturing liquid are pumped into the formation^1,2^. Formation damage^3–5^ and production rate post-fracturing^6^ are strongly related to water transport in formation. Spontaneous imbibition is one of the dominant mechanisms of water transport, especially in shale, because micro-nano pores can cause high capillary pressure^7–10^, which is a dominant force that drives wetting liquid imbibed into micro-nano pores.

The fundamental element of complex pore space is tortuous capillaries. In the early 20th century, the parallel tube bundle model was developed to represent the pore space of porous media. Fatt^11^ revised bundle of tubes as the network of tubes, which is more closely representing real porous media. Fischer *et al*.^12^ introduced the capillary pressure curve in the pore network model and studied the relationship between confining pressure and relative permeability. Bryant *et al*.^13,14^ introduced stress deformation into the pore network model to study the effect of confining pressure on permeability. Meniscus rises in capillaries comprise the basic of imbibition in porous media. Since Lucas and Washburn^15,16^ summarised the basic principle of liquid rising in a vertical capillary under the drive of capillary force and restraint of gravity, called the Lucas–Washburn (LW) equation, numerous studies have emerged over the past century. Cupelli *et al*.^17^ concluded that the inertia of liquid should be considered. Fries and Dreyer^18^ conducted a research on the analytic solution of capillary rise in an inclined tube. Kim and Whitesides^19^ studied capillary rise in noncircular capillaries. Yu^20,21^ introduced the fractal method to describe the tortuousness of capillary. Cai^22–24^ introduced fractal geometry to modify the LW equation in a single tortuous capillary tube and porous media and found that the rise time exponent is related to tortuosity fractal dimension. Li *et al*.^25,26^ studied meniscus rise in branch-like capillaries and tree-like network systems, whereas Shen *et al*.^27^ further applied this concept in shale analysis. Meng *et al*.^28^ studied mechanisms of spontaneous imbibition and the impact of boundary condition, fluid viscosity and wettability. Shou *et al*.^29^ studied geometry-induced asymmetric capillary flowing behaviours, such as multi-section porous layers and trapezoidal porous media.

In micro-nano tubes, dynamic contact angle and slip conditions are two critical characteristics. Martic *et al*.^30^ showed that the contact angle in the LW equation should be dynamic. Contact angle is related to flow velocity^31–33^. Several dynamic contact angle models have been derived. One of the most common is the power law model^34,35^, which is recommended and used to simulate experimental data^36^. Another one is the power series model, which was derived by Blake *et al*.^37^. Hilpert^38–42^ derived (semi)-analytical solutions to liquid infiltration into horizontal, infiltration and downward tubes which considers the dynamic contact angle based on the power law and series models. Meakin *et al*.^43^ summarized multiphase fluid flow and reactive transport in fractured and porous media including complete dynamic behaviour of contact lines and contact angles. Petrov *et al*.^44^ formulated qualitative criteria for applicability of hydrodynamic, molecular-kinetic and molecular-hydrodynamic theories on the basis of the wetting-dewetting asymmetry of the dynamic contact angle. Slippage in micronanopores is another important characteristic. Gas slip is obvious and well-studied in both mechanism and application in gas production^45–48^. Liquid also slip in nanopores. Several authors^49–51^ have shown that liquid infiltration in micronanopores is significantly different from the ordinary no-slip boundary. Commonly, non-wetting surfaces tend to cause non-wetting liquid slip^52,53^. Wu *et al*.^54,55^ established a universal model for water flow through nanopores, flow capacity of confined water is 10^−1^~10^7^ times that calculated by the no-slip Hagen–Poiseuille equation for nanopores with various contact angles and dimensions^54^. Sometimes wetting surfaces also have slip effect, proved by both experiment^56–58^ and molecular dynamic(MD) simulations^59^. Javadpour and Afsharpoor *et al*.^60–62^ uncovered the influence of the slip effect on the infiltration of micronanopores.

A phase portrait is a geometric representation of the trajectories of a dynamical system in the phase plane. Phase portrait analysis is a remarkable tool for analysing the dynamic process of a complex system, such as spontaneous imbibition. The phase portrait method can evaluate the dynamic process of the system without solving the complex governing ordinary differential equations (ODEs). For mechanical systems, the phase space usually consists of all possible values of position and momentum variables. The concept of phase space was developed in the late 19th century by Ludwig Boltzmann, Henri Poincaré and Willard Gibbs^63^ and is commonly used to analyse the possible state of a dynamical system.

## Spontaneous Imbibition Model

Spontaneous imbibition occurs in capillaries as wetting fluid imbibed in capillaries. This process is driven by capillary force automatically. Liquid slippage in nanoscale capillaries should also be considered. Commonly, hydrophobic surfaces tend to cause water slip^52,53^. But hydrophobic nanopores will not lead imbibition, which is not the focus of our work. We just focused on the imbibition process in this work. In weak hydrophilic nanopores, water slip also occurs. Wu *et al*.^54,55^ established a universal model for water flow through nanopores, effective slip is a linear sum of true slip and apparent slip. True slip is always positive. For water in hydrophobic tube, apparent slip is positive, while for water in hydrophilic tube, apparent slip is negative. Similar with Wu’s model, in weak hydrophilic nanopores, wettability will give slight negative effect on effective slip, but not change effective slip into negative. This is what we discussed in this model. In strong hydrophilic tube, the tube wall will induce and locked layers of water molecule, which is a multilayer sticking model with no-slip boundary^54^. Our model for slip boundary is not suitable.

Also, contact angle changes with imbibition speed which makes the imbibition process additionally complex. This section shows the establishment of a spontaneous imbibition model while considering liquid slippage and contact angle variation.

Spontaneous imbibition only exists in wetting tube, only wetting phase can induce imbibition. For inorganic pores, imbibing wetting phase is water, while for organic pores, imbibing wetting phase is oil. To simplify the investigating object and focus on the relationship between the dynamic angle and slip length, we simplify the model as wetting phase liquid imbibed in weak wetting single tube with fractal tortuosity. Certain simplifications are made. a) The cross section of the tube is circular, b) the liquid is in the wetting phase, and gas is nonwetting, c) the liquid is Newtonian with laminar flow(Inertial forces are ignored), d) the driving force of spontaneous imbibition is capillary force and e) slip occurs at the tube wall, f) Tube wall is weak wetting for liquid.

**Figure 1:**
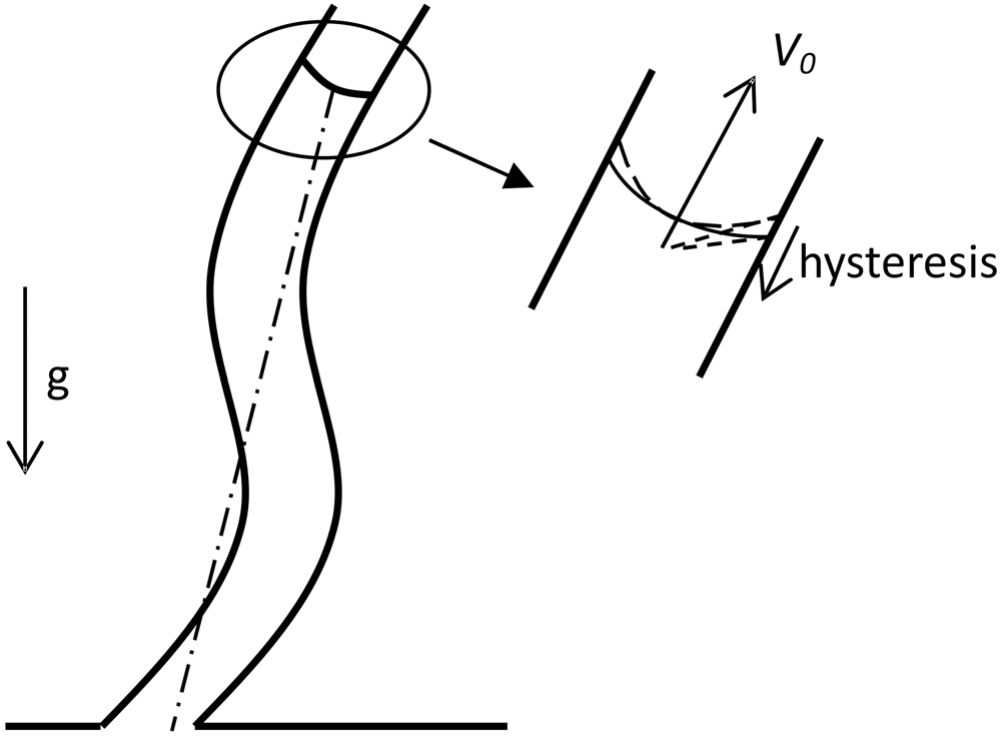
Schematic diagram of model.

Figure 1 illustrates a schematic diagram of spontaneous imbibition in fractal tortuous capillaries. In the nanoscale system, slip length *L*_*s*_ is comparable to capillary diameter. Hence, the slip effect should be considered. Velocity distribution on the cross section also follows a parabolic curve, assuming that a slip length *L*_*s*_ exists. Liquid slippage can greatly increase liquid flux. On the basis of the Hagen–Poiseuille equation, fluid flux considering liquid slippage is as follows:

1 $$Q=\frac{\pi {\rm{\Delta }}p}{2\mu {L}_{f}}[\frac{1}{2}{(R+{L}_{s})}^{2}{R}^{2}-\frac{1}{4}{R}^{4}],$$

where *Q* is fluid flux in the tube, Δ*p* is the pressure difference of the fluid, *μ* is dynamic viscosity, *L*_*f*_ is the length of tube based on liquid pathline, *R* is the equivalent radius of the tube and *L*_*s*_ is the slip length. Oftentimes, hydraulic diameter is introduced to non-circular tubes as follows:

2 $$\lambda =\frac{4{A}_{c}}{C},$$

where *λ* is the hydraulic diameter, *A*_*c*_ is the cross-sectional area and *C* is the wetted perimeter. For circular tubes, *λ* denotes the diameter or *λ* = 2*R*.

Δ*p* represents the driving pressure. For upward flow, meniscus height increases during the imbibition process.

3 $${\rm{\Delta }}p={p}_{c}-\rho g{L}_{0}\,\sin \,{\rm{\Psi }},$$

where *L*_0_ is the distance between meniscus and tube intake. For straight and vertical tubes, *L*_0_ is the height of meniscus, *ρ* is the liquid density, *g* is the gravitational acceleration, Ψ is the angle between the horizon and the tube and *p*_*c*_ is the main driving force. According to the Young–Laplace equation, *p*_*c*_ is as follows:

4 $${p}_{c}=\frac{2\sigma \,\cos \,\theta }{r},$$

where *σ* is the interfacial tension between the gas and the liquid, *θ* is the contact angle and *r* is the tube radius. In non-circular tubes, *p*_*c*_ is expressed as follows:

5 $${p}_{c}=\frac{4\sigma \,\cos \,\theta }{{D}_{h}}=\frac{4\sigma \,\cos \,\theta }{\lambda }.$$

During the imbibition process, the contact angle is dynamic. We select the liner relationship here. According to Hilpert^42^, contact angle *θ* in the Young–Laplace equation should be dynamic and expressed as follows:

6 $$\cos \,\theta -\,\cos \,{\theta }_{eq}=\tilde{\alpha }{\rm{Ca}}=-\tilde{\alpha }\frac{d{L}_{f}}{dt}\frac{\mu }{\sigma },$$

where *θ*_*eq*_ is the equilibrium contact angle and $\tilde{\alpha }\ge 0$ is a non-dimensional parameter. *Ca* is a capillary number. This model, as Hilpert^42^ assumed, is a special case of the power law and series models. Hence, *p*_*c*_ for dynamic angle can be expressed as follows:

7 $${p}_{c}=\frac{4\sigma \,\cos \,\theta }{{D}_{h}}=\frac{4\sigma }{\lambda }(\cos \,{\theta }_{eq}-\tilde{\alpha }\frac{d{L}_{f}}{dt}\frac{\mu }{\sigma }).$$

Liquid flux *Q* considering slippage, dynamic contact angle and gravity can be expressed as follows:

8 $$Q=\frac{\pi }{32\mu {L}_{f}}[\frac{1}{2}{(\lambda +2{L}_{s})}^{2}{\lambda }^{2}-\frac{1}{4}{\lambda }^{4}][\frac{4\sigma }{\lambda }(\cos \,{\theta }_{eq}-\tilde{\alpha }\frac{d{L}_{f}}{dt}\frac{\mu }{\sigma })-\rho g{L}_{0}\,\sin \,{\rm{\Psi }}].$$

Imbibition velocity is determined as follows:

9 $${v}_{f}=\frac{4Q}{\pi {\lambda }^{2}}=\frac{{\rm{d}}{L}_{f}}{{\rm{d}}t}.$$

By combining Eqs (8) and (9), we obtain

10 $${v}_{f}=\frac{{\rm{d}}{L}_{f}}{{\rm{d}}t}=\frac{1}{8\mu {L}_{f}}[\frac{1}{2}{(\lambda +2{L}_{s})}^{2}-\frac{1}{4}{\lambda }^{2}]\,[\frac{4\sigma }{\lambda }(\cos \,{\theta }_{eq}-\tilde{\alpha }\frac{d{L}_{f}}{dt}\frac{\mu }{\sigma })-\rho g{L}_{0}\,\sin \,{\rm{\Psi }}].$$

Capillaries are not always straight. Thus, tortuosity should be considered. Yu and Cheng^20^ introduced fractal dimension to express tortuosity and expressed the relationship between *L*_*f*_ and *L*_0_ as follows:

11 $${L}_{f}={\lambda }^{1-{D}_{T}}{L}_{0}^{{D}_{T}}.$$

where *L*_*f*_ is the length of fluid pathline or real length of the capillary and *L*_0_ is the linear distance between the meniscus and tube intake. Derived by Cai *et al*.^22^, the relationship between velocity *v*_*f*_ and linear velocity *v*_*C*_ can be expressed as follows:

12 $${v}_{f}=\frac{d{L}_{f}}{dt}={D}_{T}{\lambda }^{1-{D}_{T}}{L}_{0}^{{D}_{T}-1}{v}_{0},$$

where *λ* is the capillary diameter, *D*_*T*_ is the fractal dimension of a tortuous capillary and *D*_*T*_ ranges from 1 to 3. For a straight capillary *D*_*T*_ = 1, a higher *D*_*T*_ represents higher tortuosity and longer pathline capillary.

By combining Eqs (10) and (12), we can derive the ODE of imbibition as follows:

13 $${v}_{0}=\frac{{\rm{d}}{L}_{0}}{{\rm{d}}t}=\frac{[{(\lambda +2{L}_{s})}^{2}/2-{\lambda }^{2}/4]}{8\mu {D}_{T}{\lambda }^{2-2{D}_{T}}{L}_{0}^{2{D}_{T}-1}}[\frac{4\sigma }{\lambda }(\cos \,{\theta }_{eq}-\tilde{\alpha }\frac{\mu }{\sigma }{D}_{T}{\lambda }^{1-{D}_{T}}{L}_{0}^{{D}_{T}-1}\frac{{\rm{d}}{L}_{0}}{{\rm{d}}t})-\rho g{L}_{0}\,\sin \,{\rm{\Psi }}].$$

Equation (13) is a nonlinear implicit equation with independent variable *t* and dependent variables *L*_0_ and ${\dot{L}}_{0}=d{L}_{0}/dt$. The differential expression is as follows:

14 $$\frac{{\rm{d}}{L}_{0}}{{\rm{d}}t}=\frac{\frac{4\sigma }{\lambda }\,\cos \,{\theta }_{eq}-\rho g{L}_{0}\,\sin \,{\rm{\Psi }}}{\frac{8\mu {D}_{T}{\lambda }^{2-2{D}_{T}}{L}_{0}^{2{D}_{T}-1}}{[{(\lambda +2{L}_{s})}^{2}/2-{\lambda }^{2}/4]}+\tilde{\alpha }\frac{4\sigma }{\lambda }\frac{\mu }{\sigma }{D}_{T}{\lambda }^{1-{D}_{T}}{L}_{0}^{{D}_{T}-1}}.$$

At *t* = 0, the imbibition length is 0. Hence, the initial condition is as follows:

15 $${L}_{0}{|}_{t=0}=0.$$

By introducing dimensionless groups, the implicit ODE can be simplified as a dimensionless form to make the generalised equation applicable in different scales, which is helpful in performing sensitivity analyses. On the basis of the dimensionless method by Hilpert^42^, the three independent dimensionless groups are as follows:

16 $${L}_{D}=\frac{{L}_{0}}{\lambda },\,{t}_{D}=\frac{t\sigma }{\mu \lambda }\,\,{\rm{and}}\,{L}_{sD}=\frac{{L}_{s}}{\lambda },$$

where *L*_*D*_ is the dimensionless imbibition length, *t*_*D*_ is the dimensionless time and *L*_*sD*_ is the dimensionless slip length. On the basis of this dimensionless method, dimensionless velocity can be expressed as follows:

17 $$\frac{d{L}_{D}}{d{t}_{D}}=\frac{\mu }{\sigma }\frac{d{L}_{0}}{dt}.$$

We can non-dimensionalise the imbibition speed as follows:

18 $${v}_{D}=\frac{{\rm{d}}{L}_{D}}{{\rm{d}}{t}_{D}}=\frac{[{(1+2{L}_{sD})}^{2}/2-1/4]}{2{D}_{T}{L}_{D}^{2{D}_{T}-1}}(\cos \,{\theta }_{eq}-\tilde{\alpha }{D}_{T}{L}_{D}^{{D}_{T}-1}\frac{{\rm{d}}{L}_{D}}{{\rm{d}}{t}_{D}}-\frac{\rho g{\lambda }^{2}{L}_{D}\,\sin \,{\rm{\Psi }}}{4\sigma }).$$

Equation (18) remains an implicit ODE. By rearranging the first-order term, Eq. (18) can be expressed as follows:

19 $$\frac{{\rm{d}}{L}_{D}}{{\rm{d}}{t}_{D}}={\dot{L}}_{D}=\frac{\cos \,{\theta }_{eq}-\frac{\rho g{\lambda }^{2}{L}_{D}\,\sin \,{\rm{\Psi }}}{4\sigma }}{\frac{2{D}_{T}{L}_{D}^{2{D}_{T}-1}}{[{(1+2{L}_{sD})}^{2}/2-1/4]}+\tilde{\alpha }{D}_{T}{L}_{D}^{{D}_{T}-1}}$$

or simplified as follows:

20 $${\dot{L}}_{D}=\frac{A-B{L}_{D}}{C{L}_{D}^{2{D}_{T}-1}+D{L}_{D}^{{D}_{T}-1}},$$

where constants

21 $$A=\,\cos \,{\theta }_{eq},\,B=\frac{\rho g{\lambda }^{2}\,\sin \,{\rm{\Psi }}}{4\sigma },\,C=\frac{2{D}_{T}}{[{(1+2{L}_{sD})}^{2}/2-1/4]}\,{\rm{and}}\,D=\tilde{\alpha }{D}_{T}.$$

Equation (20) is an implicit first-order nonlinear ODE that represents the imbibition distance with respect to imbibition time considering liquid slip and dynamic contact angle. At initial condition *t*_*D*_ = 0, the initial interface position is at the pressure intake. Hence, dimensionless imbibition length is expressed as follows:

22 $${L}_{D}{|}_{{t}_{D}=0}=0.$$

Analytical or numerical solutions are easy to obtain. In Eq. (18), the concept that the capillary force item should be always larger than gravity item should be stressed. When the capillary force is balanced against gravity, the meniscus reaches the equilibrium height, which is expressed as follows:

$${L}_{Dend}=\frac{4\sigma \,\cos \,{\theta }_{eq}}{\rho g{\lambda }^{2}\,\sin \,{\rm{\Psi }}}.$$

## Solutions

### Initial stage of imbibition or horizontal flow

In the case of horizontal flow Ψ = 0 or at the first stage of the imbibition process, capillary force in the nanoscale tube is much larger than that in the gravity item. The gravity item can thus be neglected. Hence, after ignoring the linear term $\frac{\rho g{\lambda }^{2}\,\sin \,{\rm{\Psi }}}{4\sigma }{L}_{D}$, the item can be simplified as follows:

23 $$\frac{{\rm{d}}{L}_{D}}{{\rm{d}}{t}_{D}}=\frac{\cos \,{\theta }_{eq}}{\frac{2{D}_{T}{L}_{D}^{2{D}_{T}-1}}{[{(1+2{L}_{sD})}^{2}/2-1/4]}+\tilde{\alpha }{D}_{T}{L}_{D}^{{D}_{T}-1}}.$$

The equation is easily solved by separating the variables and subsequently implementing an integration method to derive an analytical solution. Taking the initial condition ${L}_{D}{|}_{{t}_{D}=0}=0$, the analytical solution is as follows:

24 $${L}_{D}={({\{[\frac{{(1+2{L}_{sD})}^{2}}{2}-\frac{1}{4}]t\cos {\theta }_{eq}+\frac{{\tilde{\alpha }}^{2}{[{(1+2{L}_{sD})}^{2}/2-1/4]}^{2}}{4}\}}^{\frac{1}{2}}-\frac{\tilde{\alpha }[{(1+2{L}_{sD})}^{2}/2-1/4]}{2})}^{\frac{1}{{D}_{T}}}.$$

### Gravity-influenced flow

For long-time imbibition, the influence of gravity cannot be ignored as the height of imbibition meniscus increases. The gravity term acts as a secular term. Equation (19) is an implicit ODE, which means that it cannot be typically solved. Under special circumstances (*D*_*T*_ = 1, 2), Eq. (19) has an analytical solution.

For *D*_*T*_ = 1, we can invert Eq. (19) as follows:

25 $${\dot{L}}_{D}=\frac{\cos \,{\theta }_{eq}-\frac{\rho g{\lambda }^{2}{L}_{D}\,\sin \,{\rm{\Psi }}}{4\sigma }}{\frac{2{D}_{T}{L}_{D}}{[{(1+2{L}_{sD})}^{2}/2-1/4]}+\tilde{\alpha }{D}_{T}}.$$

Under the initial condition ${L}_{D}{|}_{{t}_{D}=0}=0$ and solved by the separation of variables method, the analytical solution is as follows:

26 $$\begin{array}{rcl}t & = & \frac{2{D}_{T}}{[{(1+2{L}_{sD})}^{2}/2-1/4]}{(\frac{4\sigma }{\rho g{\lambda }^{2}\sin \Psi })}^{2}(\cos \,{\theta }_{eq}-\frac{\rho g{\lambda }^{2}\,\sin \,{\rm{\Psi }}}{4\sigma }{L}_{D})\\  &  & -\,{(\frac{4\sigma }{\rho g{\lambda }^{2}\sin {\rm{\Psi }}})}^{2}\{\frac{2{D}_{T}\,\cos \,{\theta }_{eq}}{[{(1+2{L}_{sD})}^{2}/2-1/4]}+\frac{\rho g{\lambda }^{2}\,\sin \,{\rm{\Psi }}}{4\sigma }\tilde{\alpha }{D}_{T}\}\mathrm{ln}|\cos \,{\theta }_{eq}-\frac{\rho g{\lambda }^{2}\,\sin \,{\rm{\Psi }}}{4\sigma }{L}_{D}|\\  &  & -\,\frac{2{D}_{T}\,\cos \,{\theta }_{eq}}{[{(1+2{L}_{sD})}^{2}/2-1/4]}{(\frac{4\sigma }{\rho g{\lambda }^{2}\sin {\rm{\Psi }}})}^{2}+{(\frac{4\sigma }{\rho g{\lambda }^{2}\sin {\rm{\Psi }}})}^{2}\{\frac{2{D}_{T}\,\cos \,{\theta }_{eq}}{[{(1+2{L}_{sD})}^{2}/2-1/4]}+\frac{\rho g{\lambda }^{2}\,\sin \,{\rm{\Psi }}}{4\sigma }\tilde{\alpha }{D}_{T}\}\mathrm{ln}|\cos \,{\theta }_{eq}|\end{array}$$

or

$$t=\frac{C(A-B{L}_{D})}{{B}^{2}}-\frac{AC}{{B}^{2}}\,\mathrm{ln}|A-B{L}_{D}|-\frac{D}{B}\,\mathrm{ln}|A-B{L}_{D}|-\frac{CA}{{B}^{2}}+\frac{AC+BD}{{B}^{2}}\,\mathrm{ln}|A|,$$

where constants $A=\,\cos \,{\theta }_{eq}$, $B=\frac{\rho g{\lambda }^{2}\,\sin \,{\rm{\Psi }}}{4\sigma }$, $C=\frac{2{D}_{T}}{[{(1+2{L}_{sD})}^{2}/2-1/4]}$ and $D=\tilde{\alpha }{D}_{T}$_._

For *D*_*T*_ = 2, we can invert Eq. (19) as follows:

27 $$\frac{{\rm{d}}{L}_{D}}{{\rm{d}}{t}_{D}}=\frac{\cos \,{\theta }_{eq}-\frac{\rho g{\lambda }^{2}{L}_{D}\,\sin \,{\rm{\Psi }}}{4\sigma }}{\frac{2{D}_{T}{L}_{D}^{3}}{[{(1+2{L}_{sD})}^{2}/2-1/4]}+\tilde{\alpha }{D}_{T}{L}_{D}}.$$

Setting constants $A=\,\cos \,{\theta }_{eq}$, $B=\frac{\rho g{\lambda }^{2}\,\sin \,{\rm{\Psi }}}{4\sigma }$, $C=\frac{2{D}_{T}}{[{(1+2{L}_{sD})}^{2}/2-1/4]}$ and $D=\tilde{\alpha }{D}_{T}$, Eq. (27) can be expressed as:

28 $$\frac{{\rm{d}}{L}_{D}}{{\rm{d}}{t}_{D}}=\frac{A-B{L}_{D}}{C{L}_{D}^{3}+D{L}_{D}}$$

Under the initial condition ${L}_{D}{|}_{{t}_{D}=0}=0$, it can also be solved by the separation of variables method. The analytical solution can be expressed as follows:

29 $$t=\frac{{A}^{2}(AC+BD)}{{B}^{4}}(\mathrm{ln}|A|-\,\mathrm{ln}|A-B{L}_{D}|)-\frac{C}{3B}{L}_{D}^{3}-\frac{AC}{2{B}^{2}}{L}_{D}^{2}-\frac{{A}^{2}C+{B}^{2}D}{{B}^{3}}{L}_{D},$$

For other occasions, *D*_*T*_ is not an integer. To the best of our knowledge, deriving the analytical solution is difficult. As the standard differential form has been derived as that in Eq. (19), the implicit ODE can be solved using a numerical method. We use the variable-step fourth–fifth-order Runge–Kutta algorithm (ODE45). This method is an adaptive-step numerical analysis process for ODEs and effective in obtaining the numerical solutions.

### Summary of solutions

Under different occurrences, we derived analytical and numerical solutions for spontaneous imbibition in fractal tortuous capillaries while considering dynamic contact angle and slip effect. Together with cases of gravity-ignored and -included processes and three categories of *D*_*T*_, we summarise the solutions. Table 1 illustrates the analytical and numerical solutions that we derived. Expressions of imbibition speed and length are included.

**Table 1:** Summary of solutions for imbibition speed and length (with initial condition ${L}_{D}{|}_{{t}_{D}=0}=0$).

Illustrations	Conditions	Imbibition speed	Imbibition length
horizontal flow	Ψ = 0	${v}_{D}({t}_{D})=\frac{\cos \,{\theta }_{eq}}{\frac{2{D}_{T}{L}_{D}^{2{D}_{T}-1}}{[{(1+2{L}_{sD})}^{2/2-1/4}]}+\tilde{\alpha }{D}_{T}{L}_{D}^{{D}_{T}-1}}$	$$\begin{array}{c}{L}_{D}=(\{[\frac{{(1+2{L}_{sD})}^{2}}{2}-\frac{1}{4}]t\,\cos \,{\theta }_{eq}\\ +\frac{{\tilde{\alpha }}^{2}{[{(1+2{L}_{sD})}^{2}/2-1/4]}^{2}}{4}{\}}^{\frac{1}{2}}\\ -\frac{\tilde{\alpha }[{(1+2{L}_{sD})}^{2}/2-1/4]}{2}{)}^{\frac{1}{{D}_{T}}}\end{array}$$ (analytical solution)
initial stage of imbibition	${p}_{c} > > \rho g{L}_{0}\,\sin \,{\rm{\Psi }}$		
Gravity included	*D*_*T*_ = 1	${v}_{D}({t}_{D})=\frac{\cos \,{\theta }_{eq}-\frac{\rho g{\lambda }^{2}{L}_{D}\,\sin \,{\rm{\Psi }}}{4\sigma }}{\frac{2{D}_{T}{L}_{D}}{[{(1+2{L}_{sD})}^{2/2-1/4}]}+\tilde{\alpha }{D}_{T}}$	$$\begin{array}{c}t=\frac{C(A-B{L}_{D})}{{B}^{2}}-\frac{AC}{{B}^{2}}\,\mathrm{ln}|A-B{L}_{D}|\\ -\frac{D}{B}\,\mathrm{ln}|A-B{L}_{D}|-\frac{CA}{{B}^{2}}+\frac{AC+BD}{{B}^{2}}\,\mathrm{ln}|A|\end{array}$$ (analytical solution with implicit form)
	*D*_*T*_ = 2	${v}_{D}({t}_{D})=\frac{\cos \,{\theta }_{eq}-\frac{\rho g{\lambda }^{2}{L}_{D}\,\sin \,{\rm{\Psi }}}{4\sigma }}{\frac{2{D}_{T}{L}_{D}^{3}}{[{(1+2{L}_{sD})}^{2/2-1/4}]}+\tilde{\alpha }{D}_{T}{L}_{D}}$	$$\begin{array}{c}t=\frac{{A}^{2}(AC+BD)}{{B}^{4}}(\mathrm{ln}|A|-\,\mathrm{ln}|A-B{L}_{D}|)\\ -\frac{C}{3B}{L}_{D}^{3}-\frac{AC}{2{B}^{2}}{L}_{D}^{2}-\frac{{A}^{2}C+{B}^{2}D}{{B}^{3}}{L}_{D}\end{array}$$ (analytical solution with implicit form)
	1 < *D*_*T*_ < 3, *D*_*T*_ is non-integer	${v}_{D}({t}_{D})=\frac{\cos \,{\theta }_{eq}-\frac{\rho g{\lambda }^{2}{L}_{D}\,\sin \,{\rm{\Psi }}}{4\sigma }}{\frac{2{D}_{T}{L}_{D}^{2{D}_{T}-1}}{[{(1+2{L}_{sD})}^{2/2-1/4}]}+\tilde{\alpha }{D}_{T}{L}_{D}^{{D}_{T}-1}}$	Numerical solutions(ODE45)

## Phase Portrait Analysis

Phase portrait analysis is a significant tool for analysing the dynamic state of such a system as spontaneous imbibition. The main advantage is that the method can evaluate the system without solving the complex governing ODEs, which control complex dynamic systems. Obtaining explicit solutions that are expressible in finite terms is sometimes not easy for ODEs. A qualitative study has been conducted on differential equations in which the important characteristics of the solutions to differential equations were deduced without actually solving them^64^. In the following section, a geometrical method, the phase plane, is introduced to analyse solutions to the spontaneous imbibition process in fractal tortuous capillaries considering dynamic contact angle and slip effect.

A phase portrait is a geometric representation of the trajectories of a dynamical system in the phase plane, which consists of the system trajectories (arrows), stable steady states (dots) and unstable steady states (circles) in state space. The phase portrait method is useful in studying dynamical systems and forms a series of typical trajectories in the state space; these trajectories can reveal the stability of solutions, tendency towards asymptote and influence of initial condition disturbance on the ultimate state.

**Figure 2:**
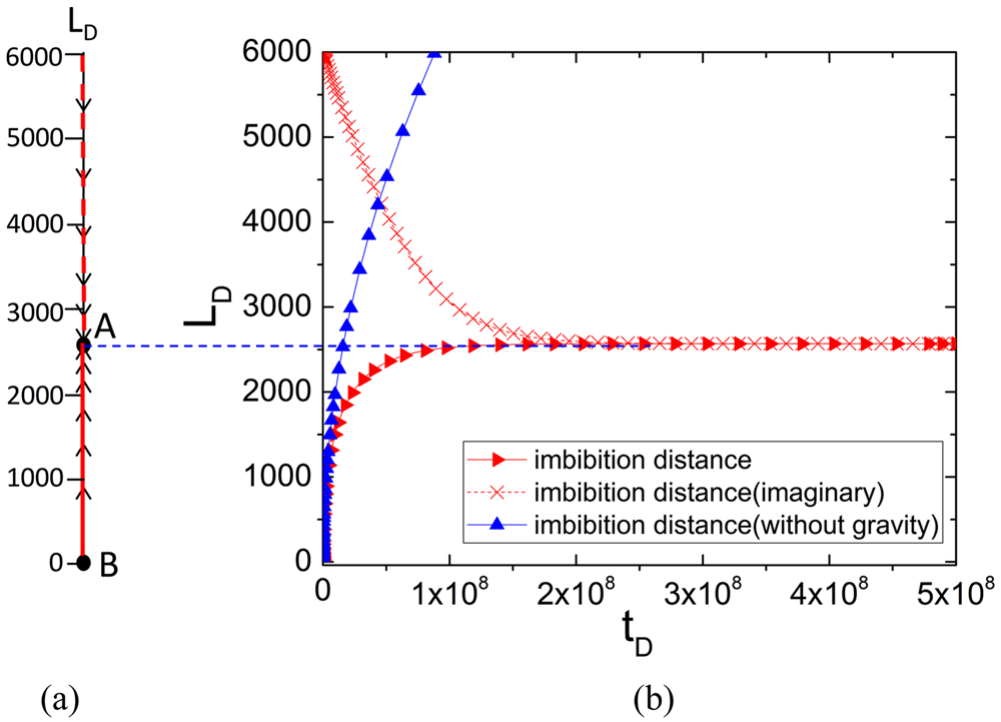
Numerical solution and phase line.

For spontaneous imbibition, we develop a series of phase portraits to analyse the stability of solutions to the imbibition model and influence of variables on the imbibition process. We first draw the phase line of spontaneous imbibition, as shown in Fig. 2(a), and then compare. We numerically solve Eq. (19) to derive the dimensionless imbibition length *L*_*D*_ versus dimensionless imbibition time *t*_*D*_ using the ODE45 method, as shown in Fig. 2(b). The negative value of *L*_*D*_ does not have physical meanings as imbibition distance *L*_*D*_ is always a positive value (*L*_*D*_ > 0). Hence, negative values are ignored. Phase line is a 1D phase system that represents an interval of the domain of the derivative in the system. Values on the line denote imbibition distance, whereas arrows mean direction of the meniscus movement. As arrow density increases, velocity of meniscus decreases. Figuratively speaking, phase line Fig. 2(a) is a projection of Fig. 2(b). At the end of the imbibition speed, capillary force is equal to the gravity of the liquid. The dynamical system becomes stable, and this stage is also called the equilibrium state. For an autonomous ODE in a single variable, indicating the critical points is easy. By setting ${\dot{L}}_{D}=0$, every point becomes a stable equilibrium because *L*_*D*_ does not change. If ${\dot{L}}_{D} > 0$, then *L*_*D*_ is always increasing for all *L*_*D*_. Furthermore, if ${\dot{L}}_{D} < 0$, then *L*_*D*_ is always decreasing. Hence, by setting ${\dot{L}}_{D}=0$ with *D*_*T*_ > 1, we have two typical points: *L*_*D*1_ = *A*/*B*, *L*_*D*2_ = 0, where *L*_*D*2_ is the extraneous root.

From the phase line, we can visually observe the stability of typical points. Point A represents the solution ${L}_{D1}=\frac{A}{B}$. Both arrows point towards critical point A, whereas the dynamic system ends in equilibrium state. We describe point A as a stable node, and the solution is stable under small perturbations. By representing *L*_*D*2_ = 0, the direction of movement deviates from point B. If the negative volumes of *L*_*D*_ are drawn in this figure, the direction of movement also deviates from point B. Hence, point B is called the unstable node in which any slight perturbation will lead to an unstable solution.

From the physical meaning of this model, spontaneous imbibition starts at ${L}_{D}{|}_{{t}_{D}=0}=0$. Figure 2(a) shows the imbibition curve as a red line. While spontaneous imbibition starts at ${L}_{D}{|}_{{t}_{D}=0}=6000$, the gravity of the liquid is larger than the capillary force and the meniscus moves downwards (supposing the contact angle equations are the same). Finally, the dynamic system becomes stable at equilibrium height. The solution is drawn as a dashed line in Fig. 2(b) because solutions to *L*_*D*_ that are larger than the equilibrium height are mainly not discussed in this model. If we set gravity as zero in this system, then the system becomes an unlimited one and *L*_*D*_ will increase without limitation (if the tube length is infinite). Hence, the system will not reach a stable state. The solution line for an unlimited system is drawn as a blue line in Fig. 2(b).

**Figure 3:**
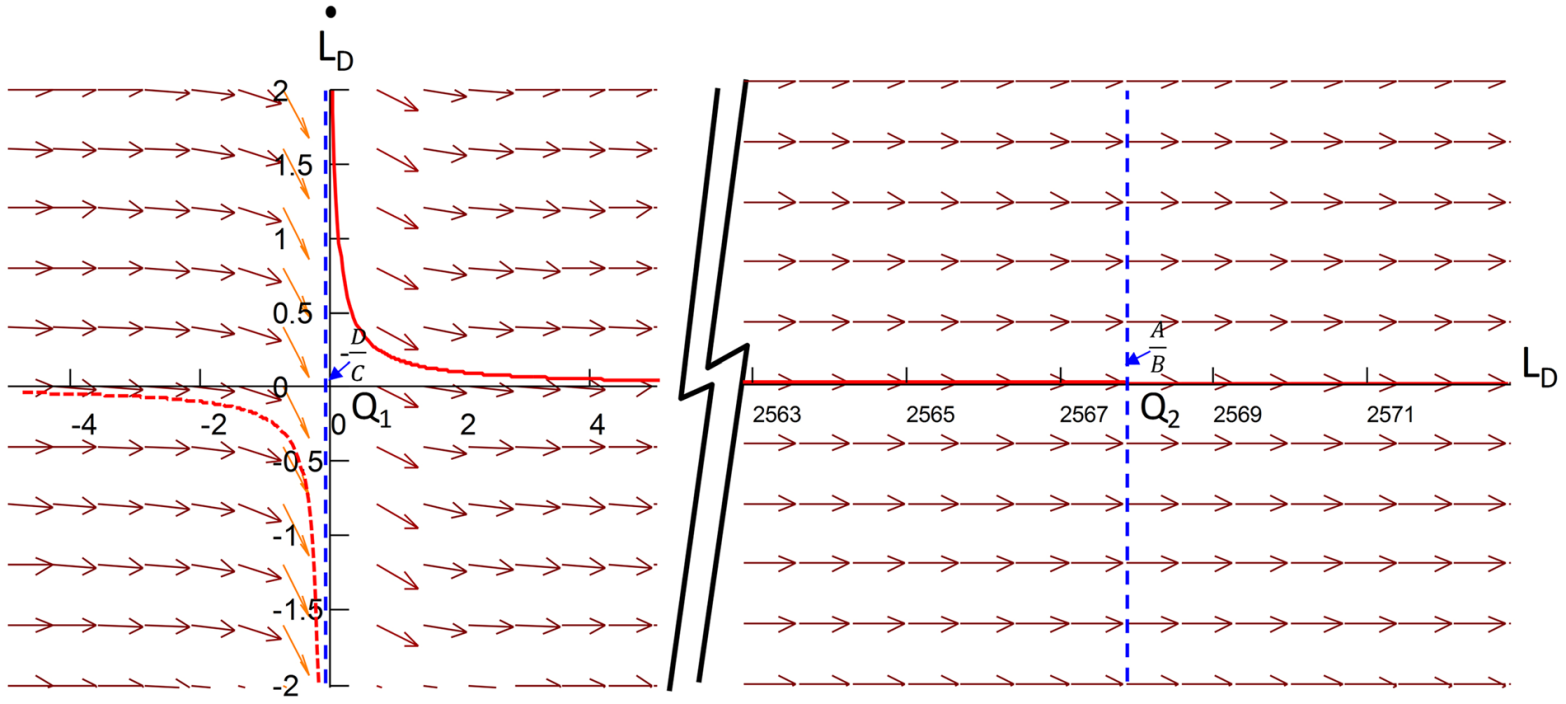
2D phase plane and vector field.

A 2D phase figure composed by a pair of values $({L}_{D},{\dot{L}}_{D})$ is called a phase plane, which gives the imbibition velocity ${\dot{L}}_{D}$ at meniscus position *L*_*D*_. The solutions to the differential equation are a family of functions. Figuratively speaking, these solutions can be drawn in phase plane filled with a vector field. Vectors represent the direction of meniscus movement. Figure 3 shows a typical 2D phase plane and vector field for the solutions to Eq. (19). Given the initial condition, the solution line in phase plane is drawn as a red line. A certain part of the coordinate *L*_*D*_ is omitted. The left vertical asymptote is *L*_*D*_ = −*D*/*C*, and the right intersection with coordinate *L*_*D*_ is *L*_*D*_ = *A*/*B*. The origin of coordinates Q1 is a saddle point because vectors around point Q1 are opposite. At point Q2, where *L*_*D*_ is larger than *A*/*B*, the velocity is a negative number. Hence, meniscus flow direction will flow back to Q2 and the dynamic system will be stable at Q2, which is a stable node. From the physical meaning of this model, the dashed line has no physical meaning, although it is a solution line. Area 0 < *L*_*D*_ < *A*/*B* is our focus.

## Model Verification

**Figure 4:**
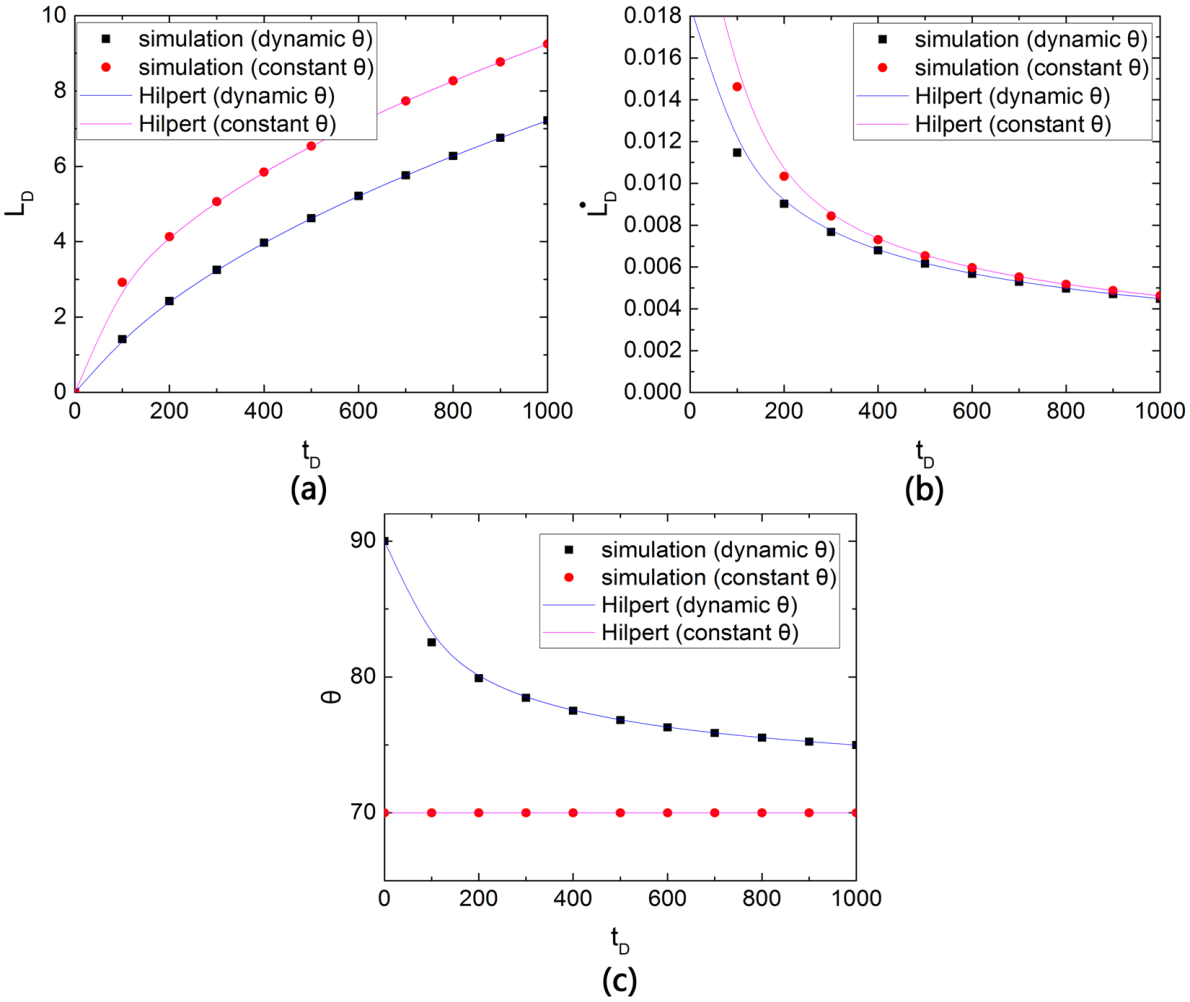
Model verification with simulation results and Hilpert’s data.

We use Hilpert’s public data^41^ of the spontaneous imbibition model to illustrate and verify our solutions for the imbibition process. Hilpert developed analytical solutions to spontaneous imbibition in capillaries without surface slip. By setting the same parameters, the solutions and published data are drawn under the same coordinate. Figure 4 shows the validation for dimensionless imbibition length and speed and dynamic contact angle.

Several physical properties of capillaries show their influence on imbibition height. Hilpert’s model is a straight capillary without surface slip. By setting *D*_*T*_ = 1 and *L*_*sD*_ = 0, Eq. (19) can be simplified as Hilpert’s model. Moreover, similar to Hilpert’s model, $\tilde{\alpha }=18.5$, static contact angle *θ*_*eq*_ = 70^0^. If contact angle remains stable in the imbibition process, then $\tilde{\alpha }=0$. If the dynamic contact angle *θ* is equal to the static contact contact angle, then *θ* = *θ*_*eq*_ = 70^0^. Numerical solutions are derived using the ODE45 method and shown as dots in Fig. 4, whereas Hilpert’s data are shown as lines. The result shows that our model coordinates well with the published data. In our work, the definition for dimensionless height and time are *L*_*D*_ = *L*_0_/*λ* and *t*_*D*_ = *tσ*/(*μλ*), whereas those of Hilpert’s are *L*_*D*_ = *L*_0_/*R* (or *L*_*D*_ = 2*L*_0_/*λ*) and *t*_*D*_ = *tσ*/(*μR*) (or *t*_*D*_ = 2*tσ*/(*μλ*)). Hence, Hilpert’s original data have a conversion process.

## Results and Discussions

### Slip length

**Figure 5:**
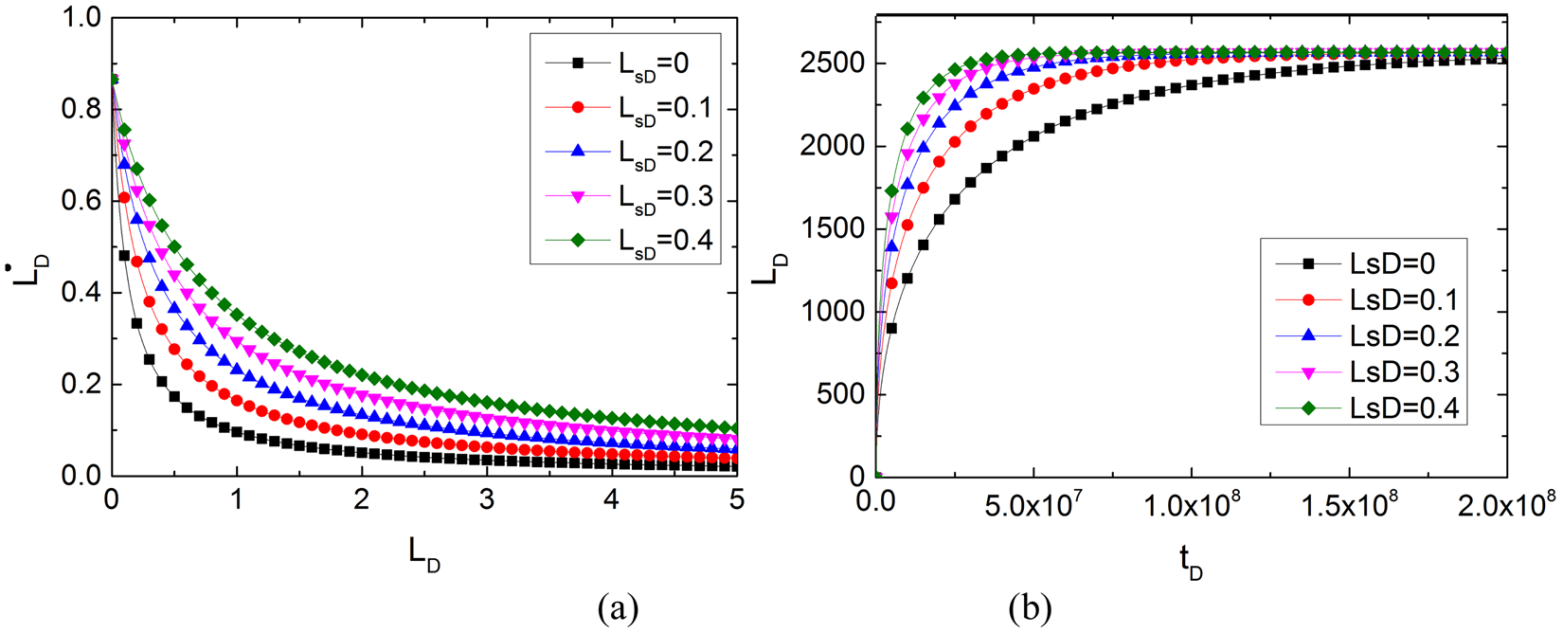
Influence of slip length on phase lines and imbibition curve.

In nanopores, the slip effect has a significant influence on the spontaneous imbibition process. Figure 5(a) shows the phase plane analysis under slip effect. When gravity is considered, the phase line extends to the X axis. By changing the dimensionless slip length of the system, a series of phase paths exists. All of the phase paths approach the X axis (${\dot{L}}_{D}=0$), which indicates that the dynamic system will be stable with any slip length. Furthermore, ${\dot{L}}_{D}=0$ is an asymptote. Imbibition speed increases with slip length. Figure 5(b) shows the dimensionless imbibition length versus imbibition time for different dimensionless slip lengths (*L*_*sD*_). As shown in this figure, the higher the *L*_*sD*_ value, the longer the imbibition at the same imbibition time. Figure 5(b) shows that at *t*_*D*_ = 2.5 × 10^7^, imbibition length *L*_*D*_ is only 1700 for no-slip cases. The system reaches stable state at *t*_*D*_ = 2 × 10^8^. For slip-influenced cases, as dimensionless slip length increases, average imbibition speed and dimensionless height increase. At *t*_*D*_ = 2.5 × 10^7^, when *L*_*sD*_ reaches 0.4, imbibition length *L*_*D*_ becomes 2500, whereas *L*_*D*_ only reaches 1700 for no-slip cases. Imbibition length *L*_*D*_ increases by 47% because of slip length.

The simulated data indicate that the slip effect has an apparent advantage on the imbibition process. Most throats in shale are in microscale and nanoscale. When analysing the spontaneous imbibition process, overlooking slippage will considerably underestimate the imbibition flux, which is special compared with sandstones. Pore space in sandstones is large, with most diameters of throats in millimetre-scale and microscale, whereas slip length mainly exists in nanoscale^62^. Under this circumstance, the value of *L*_*sD*_ is merely in a 0.001 scale, which is close to 0. Traditional imbibition models are based on imbibition analyses of sandstone, in which ignoring slippage is acceptable. Meanwhile, in imbibition analyses for shale, throats are mainly in nanoscale, which are in the same scale as slip length. Hence, dimensionless slip length *L*_*sD*_ is approximately 0.1–10. Figure 5(b) shows that slip effect in this situation noticeably and significantly promotes the imbibition process. Hence, slippage cannot be neglected in micronanopores.

### Dynamic contact angle

**Figure 6:**
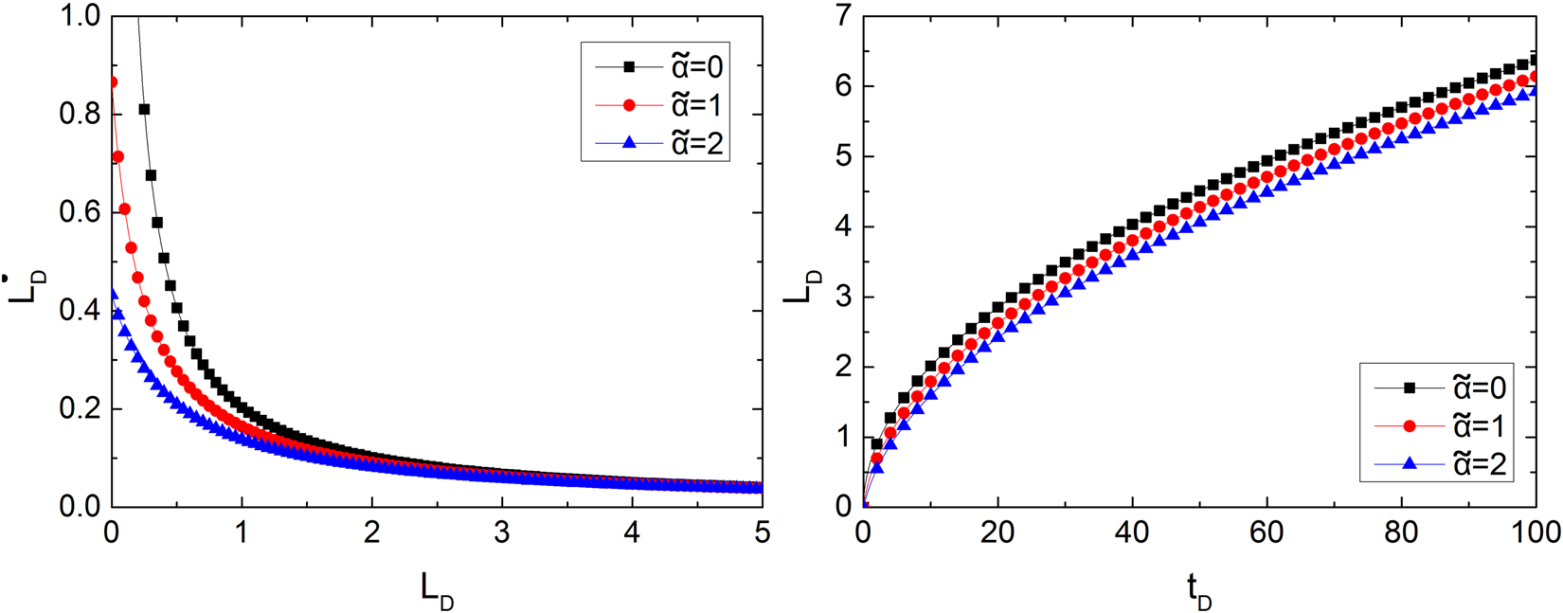
Influence of dynamic contact angle on phase lines and imbibition curve.

Apart from slippage, the dynamic contact angle has a significant influence on the imbibition process. Figure 6(a) shows the phase plane analysis of the influence of dynamic contact angle on spontaneous imbibition. When gravity is considered, the dynamic system can also reach equilibrium state because all of the phase paths point towards the equilibrium point. The dynamic contact angle is influenced by imbibition speed. Hence, capillary forces have a nonlinear relation to imbibition length. If $\tilde{\alpha }=0$, then the dynamic contact angle is equal to the static angle. As $\mathop{\alpha }\limits^{ \sim }$ increases, the effect of dynamic contact angle on imbibition process becomes larger. Figure 6(a) shows that dynamic contact angle mainly influences the initial stage of imbibition. The influence diminishes as imbibition length increases. Meanwhile, Fig. 6(b) shows the dimensionless imbibition length versus time for different dynamic coefficients ($\mathop{\alpha }\limits^{ \sim }$). $\mathop{\alpha }\limits^{ \sim }$ significantly influences the early stage of imbibition. If *t*_*D*_ > 40, then three curves are nearly parallel to one another, which indicates that their imbibition speeds are similar.

In the imbibition process, the rough flowing wall has resistance to fluid, which causes a ‘delay’ effect on the contact angle. The dynamic contact angle becomes bigger than the static one, whereas the capillary force in the dynamic process becomes smaller than that under static situation. High imbibition speed will cause a heavy ‘delay’ effect and small capillary force. At the initial stage, the imbibition speed or ${\mathop{L}\limits^{.}}_{D}$ is fast. Hence, the effect of the dynamic angle is apparent. At the end of the imbibition process, the effect of the dynamic angle is negligible.

### Gravity

**Figure 7:**
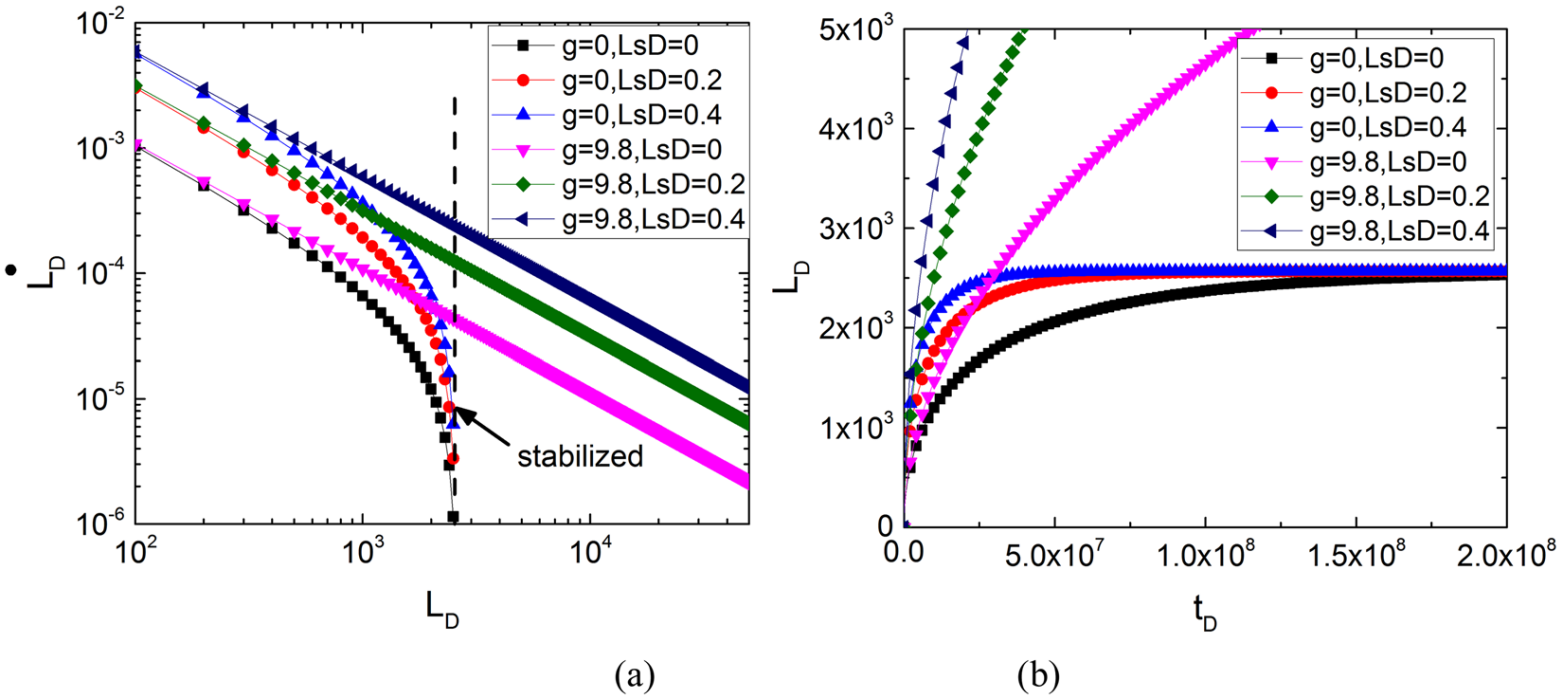
Influence of gravity on phase lines and imbibition curve.

In the previous sections, gravity is considered while the dynamic system is stable at equilibrium height. In a no-gravity environment, the dynamic system is not stable at the end. Discussing the stability of the system is easy using phase portrait analysis. Figure 7(a) shows the phase plane analysis with gravity. The phase paths that form a family of parallel straight lines are *g* = 0. The phase paths are apparently parallel straight lines that do not show any sign of convergence. Meanwhile, when gravity is considered, all phase lines converge at the stabilised line. Figure 7(b) shows the imbibition curve with and without gravity. The curve is solved using the ODE45 computation. As imbibition time increases, the gravity-considered imbibition height becomes stable and stops increasing at equilibrium height. Under the no-gravity condition, the increase in imbibition height does not stop. This result is the same as that in phase plane analysis.

**Figure 8:**
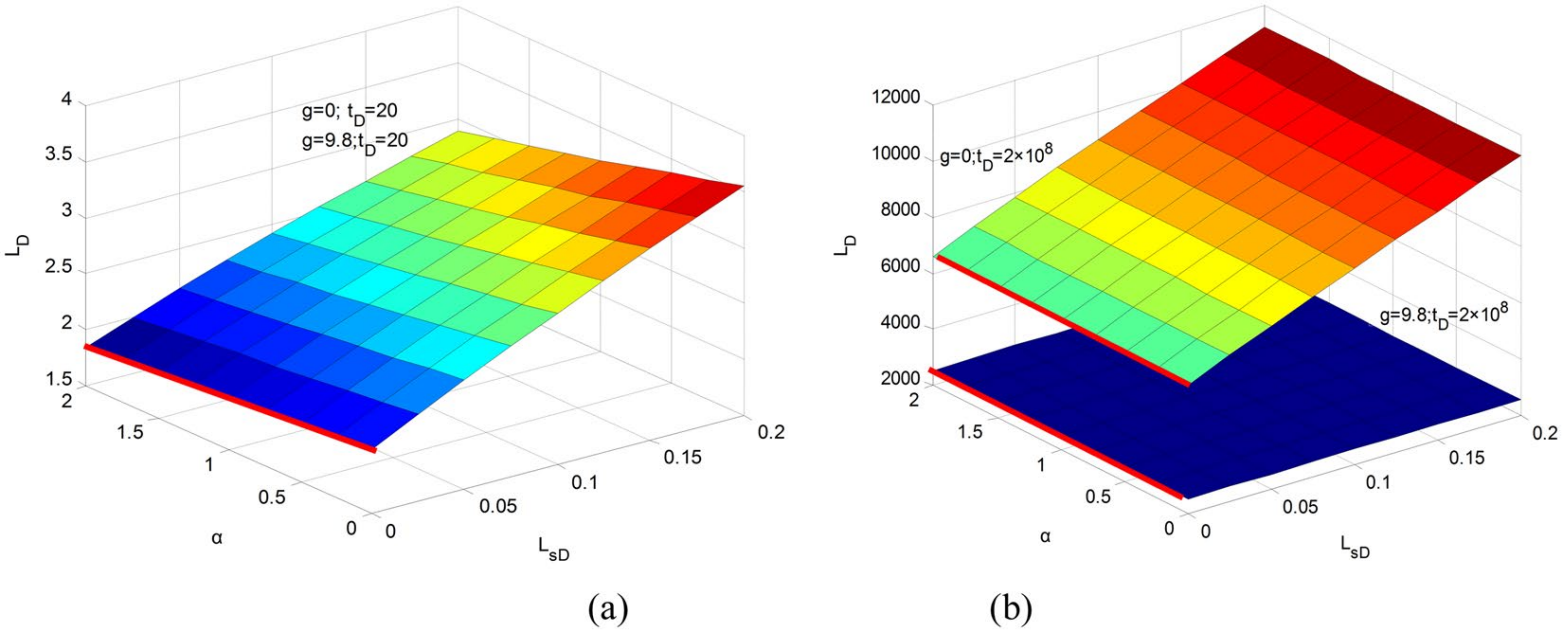
Influence of parameters on imbibition height in 3D space.

Suggesting slip length is constant. The imbibition process varies with different capillary diameters. A large capillary gains a small *L*_*sD*_. The influence of gravity is noticeable in the imbibition process. At the beginning of the process, imbibition speed is much smaller in a large capillary than that in a small capillary regardless of whether gravity is considered or not. As *L*_*D*_ increases, gravity can decrease the imbibition speed. In conclusion, gravity is one of the key factors that control the stability of this spontaneous imbibition system. The coupling relationship among gravity, dynamic contact angle and slip length is complex. To explicitly express this coupling relationship, 81 independent simulation cases that are based on the solutions to the imbibition process are performed in a 3D parameter space. Figure 8(a) and (b) show the parameter planes. Figure 8(a) depicts the parameter space at *t*_*D*_ = 20, which indicates the initial stage of the imbibition process, and Fig. 8(b) shows the parameter space at *t*_*D*_ = 2 × 10^8^, which indicates the last stage.

The figures show that at the beginning of imbibition, the two surfaces almost overlap and the influence of gravity is not large. *α* acts as a resistance, and *L*_*sD*_ acts as a promotion. Meanwhile, a small *α* and large *L*_*sD*_ characterise a positive imbibition process. The red line denotes a no-slip condition. Imbibition speed at the slip boundary is several times bigger than that at the no-slip boundary. At the end of the imbibition process, *α* and *L*_*sD*_ have nearly no effect on imbibition height because the system has reached equilibrium height without the influence of these parameters. This condition verifies the analytical solution previously discussed. Under the gravity-considered condition, *α* has no influence on imbibition height and *L*_*sD*_ also acts as a promotion.

### Typical situations and time complexity

**Figure 9:**
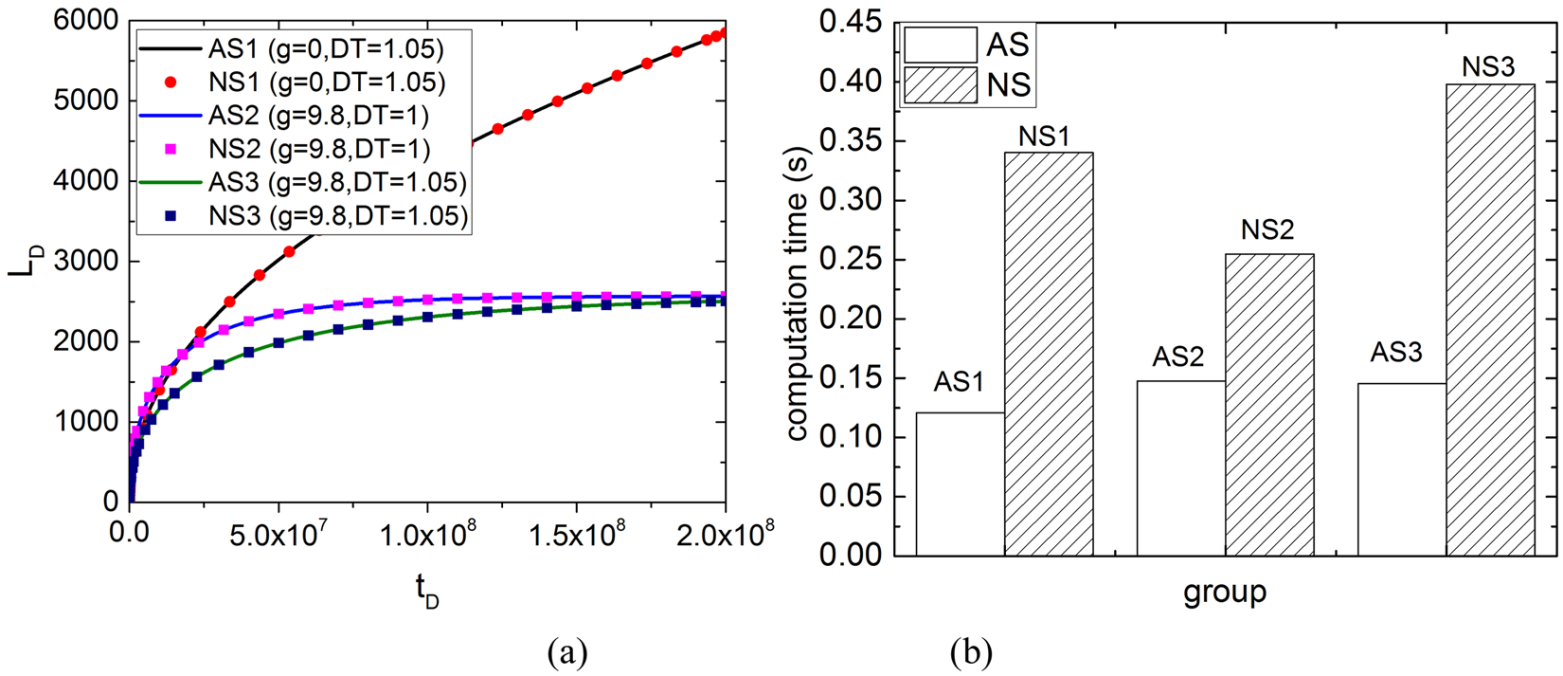
Solutions and calculation time for analytical and numerical methods. (AS: analytical solution, NS: numerical solution).

As shown in Table 1, the spontaneous imbibition function can be derived from an analytical solution under certain special situations, such as *D*_*T*_ = 1, 2, *g* = 0. Without these special situations, the imbibition function can be solved using numerical models, such as the ODE45 method, which is commonly used to solve complex ODEs. In Fig. 9(a), we compare the analytical and numerical solutions of the ODE for the imbibition process and obtain excellent agreement.

The fundamental process of the solution is iteration because ODE45 is a discretisation method. Although numerical solutions agree with the analytical solutions, running numerous iterations consumes considerable calculation time. Figure 9(b) shows the time consumed in one capillary for both solutions. Numerical solutions consume more time than analytical solutions. For certain complex ODEs, numerical methods do not easily converge during the iteration process and numerical solutions cannot be derived. For these ODEs, the phase portrait analysis method is a brilliant tool for system analysis because it does not rely on analytical solutions to complex ODEs. A large amount of calculation time can be avoided. Hence, phase portrait analysis can significantly shorten the calculation time and provide a promising way for complex dynamic system analysis.

## Conclusions

In this work, the spontaneous imbibition model in fractal tortuous capillaries is successfully built, considering dynamic contact angle and slip effect during the imbibition process. On the basis of this model, ODEs are built describing dimensionless imbibition height versus dimensionless imbibition time. We introduce phase portrait analysis to analyse the imbibition process. Furthermore, when *D*_*T*_ = 1, 2 and *g* = 0, analytical solutions for the imbibition equation are derived. Under other conditions, numerical solutions are derived. Additionally, in the imbibition model, we explore influential factors for spontaneous imbibition. The accuracy and reliability of phase portrait analysis and the analytical solutions are examined. The phase portrait analysis method is a promising way for analysing complex spontaneous imbibition. Finally, a 3D parameter space based on the imbibition model is performed to express the relationship among slip length, dynamic angle and gravity.

Findings show that phase portrait analysis is a significant tool for analysing the spontaneous imbibition process. The most prominent advantage is that the phase portrait method can evaluate the process without solving the complex governing ODEs. Phase portrait analysis and the solutions to the ODEs reveal that dynamic contact angle and slip effect play an important role in fluid imbibition in fractal tortuous capillaries. The calculations show that considering slippage in imbibition simulation significantly increases imbibition flux in nanotubes, whereas considering dynamic contact angle corrects the imbibition process at the initial stage. Finally, gravity is a key factor that controls the stabilisation of the imbibition process.
